# Identification of a New Role of miR-199a-5p as Factor Implied in Neuronal Damage: Decreasing the Expression of Its Target X-Linked Anti-Apoptotic Protein (XIAP) After SCI

**DOI:** 10.3390/ijms252212374

**Published:** 2024-11-18

**Authors:** Teresa Muñoz-Galdeano, David Reigada, Altea Soto, María Asunción Barreda-Manso, Pablo Ruíz-Amezcua, Manuel Nieto-Díaz, Rodrigo M. Maza

**Affiliations:** Molecular Neuroprotection Group, Research Unit, National Hospital for Paraplejics (SESCAM), 45071 Toledo, Spain; dreigada@sescam.jccm.es (D.R.); alteasotoneira@gmail.com (A.S.); mbarreda@sescam.jccm.es (M.A.B.-M.); prazmezcua@sescam.jccm.es (P.R.-A.); mnietod@sescam.jccm.es (M.N.-D.)

**Keywords:** spinal cord injury, apoptotic cell death, neural cells, XIAP, neuroprotection, microRNA-based therapies

## Abstract

Spinal cord injury (SCI) results in a cascade of primary and secondary damage, with apoptosis being a prominent cause of neuronal cell death. The X-linked inhibitor of apoptosis (XIAP) plays a critical role in inhibiting apoptosis, but its expression is reduced following SCI, contributing to increased neuronal vulnerability. This study investigates the regulatory role of miR-199a-5p on XIAP expression in the context of SCI. Using bioinformatic tools, luciferase reporter assays, and in vitro and in vivo models of SCI, we identified miR-199a-5p as a post-transcriptional regulator of XIAP. Overexpression of miR-199a-5p significantly reduced XIAP protein levels, although no changes were observed at the mRNA level, suggesting translational repression. In vivo, miR-199a-5p expression was upregulated at 3 and 7 days post-injury, while XIAP expression inversely decreased in both neurons and oligodendrocytes, being particularly significant in the latter at 7 dpi. These findings suggest that miR-199a-5p contributes to the downregulation of XIAP and may exacerbate neuronal apoptosis after SCI. Targeting miR-199a-5p could offer a potential therapeutic strategy to modulate XIAP levels and reduce apoptotic cell death in SCI.

## 1. Introduction

Spinal cord injury (SCI) is a multifaceted pathological condition characterized by the partial or complete loss of motor and sensory functions, imposing significant physical and social consequences for patients globally [1,2]. The cascade of damage ensuing from traumatic SCI initiates with a primary injury resulting from direct contusion, laceration, and/or compression, leading to cellular demise primarily through necrotic mechanisms. Subsequently, the injury progresses to a secondary phase that includes diverse pathological processes including excitotoxicity, oxidative stress, and heightened immune reactivity. This noxious environment leads to further structural and functional alterations that spread neural cell death spatially and temporally beyond the initial trauma site to the neighboring cells [3]. In this phase, one of the main forms of neural cell death is apoptosis, a programmed cell death highly regulated [4], promoted by both internal and external stimuli. Following primary SCI, immediate alterations occur in gene expression patterns. These expression changes affect both protein-coding genes and regulatory non-coding genes, particularly microRNAs (miRNAs), which play crucial roles in post-transcriptional regulation through modulation of mRNA stability and translation [5,6]. The miRNAs are short (19–25 nt) non-coding RNA sequences, involved in regulating various physiological and pathophysiological mechanisms following SCI. For instance, differentially expressed miRNAs can influence secondary neuroinflammation or neuronal cell death contributing to functional impairment [7,8,9,10,11,12]. Research from our group and other laboratories has shown that the dysregulation of miRNA expression is involved in the regulation of programmed cell death proteins [13,14,15]. Thus, increased miR-711 expression post-SCI correlates with the downregulation of the pro-survival protein Akt [16], whereas reductions in miR-27a may potentiate programmed cell death by enabling the expression of pro-apoptotic Bcl-2 family proteins such as Noxa, Puma, and Bax [17]. An additional antiapoptotic factor subject to microRNA-mediated regulation across diverse cell types and pathological contexts is the X-linked inhibitor of apoptosis (XIAP). As a member of the inhibitor of apoptosis (IAP) family, XIAP suppresses apoptosis by inhibiting the processing, activation, and maturation of the initiator caspase-9 and the effector caspases-3 and -7 [18,19]. While endogenous XIAP is not required for the survival of neurons under normal non-stressed conditions [20], its downregulation or knock-out makes neurons more vulnerable to multiple apoptotic triggers [21,22,23,24]. XIAP, but not other members of the IAP family such as cIAP-1 and cIAP-2-, undergoes cleavage within the initial days following SCI, which has been linked to caspase activation and increased risk of apoptosis of neural cells [25,26,27]. Moreover, overexpression of XIAP has been demonstrated to prevent neuronal cell death after SCI [28], axotomy, cerebral ischemia, and hypoxia [29]. Siegel et al. showed that downregulation of miR-23a is associated with reduced cell death after cerebral injury by increasing XIAP levels and subsequent inhibition of caspase activation [30]. The main aim of the present work is to identify miRNAs with altered expression after SCI which potentially modulate XIAP expression, employing a bioinformatic approach and subsequently investigating this interaction in a contusive SCI model. Through our analysis, we identified several miRNA candidates and validated miR-199a-5p as a post-transcriptional regulator of XIAP in vitro. Furthermore, we examined the expression changes of both miR-199a-5p and XIAP in an in vivo SCI model. These findings provide new insights into the role of miR-199a-5p in neural cell death after SCI.

## 2. Results

### 2.1. miR-199a-5p Is a Potential Regulator of XIAP Expression

**Figure 1:**
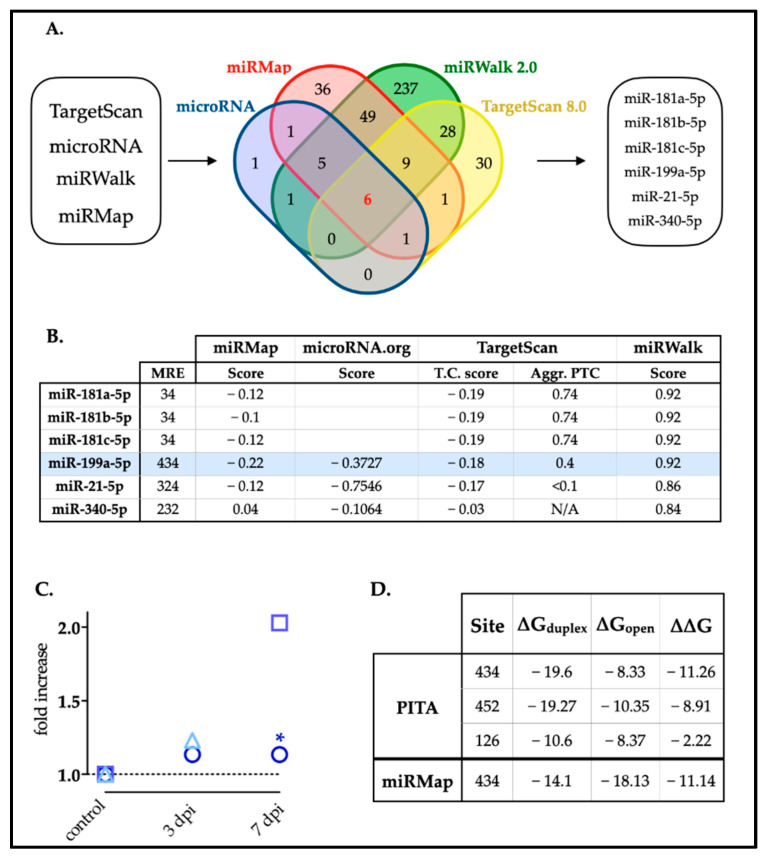
Selection of miRNAs with predicted MREs in the rat 3′-UTR-XIAP. (**A**) Venn diagram representing the number of miRNAs predicted by each of the four algorithms (TargetScan 8.0, miRanda, miRWalk and miRMap); or by more than one. Six miRNAs candidates were predicted by all four algorithms: miR-181a-5p, miR-181b-5p, miR-181c-5p, miR-199a-5p, miR-21-5p, and miR340-5p. (**B**) The table shows the selected miRNAs and the prediction scores calculated by each algorithm. miR-199a-5p is highlighted in blue. (**C**) Summary graph illustrating miR-199a-5p fold increase data at 0, 3, and 7 dpi (days post-injury) from our previous study and others. Circles represent data from Yunta et al. [6], * *p* < 0.05 at 7 dpi; squares represent data from Liu et al. [33]; and triangles represent data from Chen et al. [32]. (**D**) Localization of the different MREs for miR-199a-5p in the whole sequence of the rat 3′UTR-XIAP, indicating the ∆∆G score for miRNA–target interactions, computed as the free energy gained by transitioning from the state in which the miRNA and the target are unbound (∆G open) and the state in which the miRNA binds its target (∆G duplex), according to PITA algorithm.

Using in silico methodologies, we searched for miRNA candidates with miRNA response elements (MREs) within the mRNA sequence of rat XIAP, encompassing both the 3′UTR and 5′UTR, as well as the coding regions. Combining data obtained from four different miRNA target prediction algorithms (TargetScan, miRanda, miRWalk, and miRMap) (Figure 1A), our analysis revealed a shared prediction of six miRNAs across all four tools: miR-181a-5p, miR-181b-5p, miR-181c-5p, miR-199a-5p, miR-21-5p, and miR-340-5p. Notably, the identified MREs for these miRNAs are exclusively situated within the 3′UTR region (3′UTR-XIAP) but neither in 5′-UTR nor in the coding region. The prediction scores obtained from each algorithm indicated that miR-199a-5p, miR-181a-5p, and miR-181b-5p showed the highest scores (Figure 1B). However, given prior validations of XIAP as a target of miR-181a-5p and miR-181b-5p [31], and consistent with reports indicating upregulation of only miR-199a-5p after SCI [6,32,33] (Figure 1C, see also Supplementary Figure S1), we focused our efforts on validating miR-199a-5p as a regulator of XIAP expression. We evaluated the probability of binding miR-199a-5p to MREs within the XIAP mRNA sequence by studying their folding and accessibility scores using the miRMap and PITA algorithm (Figure 1D). The PITA algorithm suggested that miR-199a-5p have several potential MREs with favorable ∆∆Gs, particularly a site starting in the nucleotide 434 of the 3′UTR-XIAP. Specific analyses for nucleotide 940 MRE confirm its accessibility (Figure 1D), considering that the energy required to open the target mRNA secondary structure is smaller than the energy gained by the miRNA binding (∆G_open_ = −8.33 kcal/mol vs. ∆G_duplex_ = −19.6 kcal/mol) leading to a net gain of energy (∆∆G = −11.26 kcal/mol). Moreover, the minimal folding energy estimated by miRmap also predicted a stable structure for the miR-199a–XIAP duplex (∆G_total_ = 9.8 kcal/mol). Therefore, all employed algorithms suggest that microRNA-199a-5p had several different predicted MREs with highly favorable ∆∆G values (Figure 1B), particularly the one in nucleotide 434 of the 3′UTR-XIAP.

In summary, the bioinformatics analysis supports miR-199a-5p as a potential regulator of XIAP expression, suggesting that miR-199a-5p plays a key role in modulating XIAP through its interaction with the 3′UTR-XIAP sequence.

### 2.2. miR-199a Targets the 3′UTR-XIAP Sequence

To validate the effective binding of miR-199a-5p to the rat 3′UTR-XIAP, luciferase reporter assays were conducted. For this purpose, the whole wild-type (pmiRGLO^XIAP^) and mutated (pmiRGLO^XIAP-mut^) 3′UTR-XIAP were subcloned downstream of the Luciferase reporter gene in the pmiR-GLO vector. No significant alterations in luciferase activity were observed in rat C6 cells transfected with the pmiR-GLO plasmid, either with or without co-transfection with the miR-199a-5p mimic, thus ruling out any influence of endogenous miRNAs or miR-199a-5p on plasmid expression. Subsequently, C6 cells were co-transfected with either the wild-type or mutant plasmid, along with either the miR-199a-5p or the negative control mimics.

**Figure 2:**
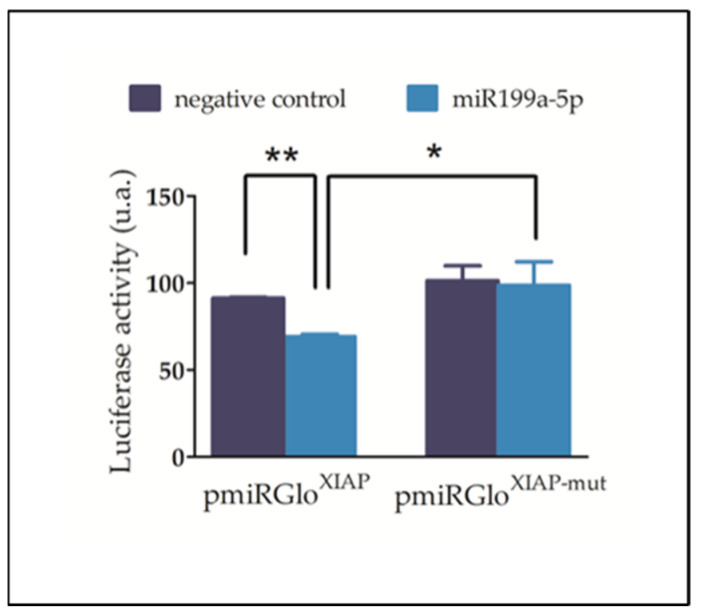
MiR-199a-5p effectively binds to the 3′-UTR-XIAP and reduces luciferase reporter gene expression. Luciferase reporter assay following co-transfection of C6 cells with either pmiRGlo-3′-UTR-XIAP (pmiRGLO^XIAP^) or pmiRGLO-3′UTR-XIAP-mut (pmiRGLO^XIAP-mut^), along with miR-199a-5p or negative control mimics. The bar graph summarizes Firefly/Renilla emission ratio normalized versus double negative control (empty pmiRGLO^0^ + negative control mimic). Bars represent mean ± SD of n = 3 independent experiments. * *p*-value > 0.05; ** *p*-value < 0.01.

As shown in Figure 2, co-transfection of miR-199a-5p mimic significantly reduced the luciferase activity of pmiRGLO^XIAP^ plasmid (34.96 ± 7.13% reduction), compared to co-transfection with the negative control mimic (Luciferase/Renilla ratios: pmiRGLO^XIAP^ + miR199a-5p mimic = 61.69 ± 4.4; pmiRGLO^XIAP^ + Neg. Ctrl mimic = 100.23 ± 12.3; t_2_ = 12.06 in paired *t*-test, *p*-value = 0.0034, n = 3). In contrast, transfection with miR-199a-5p mimic did not cause any reduction in pmiRGLO^XIAP-mut^ luciferase activity (1.0 ± 1.1%), compared to co-transfection with the negative control mimic (Luciferase/Renilla ratio: pmiRGLO^XIAP-mut^ + miR199a-5p mimic = 128.47 ± 10%; t_2_ = 0.12 in paired *t*-test, *p*-value = 0.46, n = 3), thereby confirming the specificity of miR-199a-5p regulation on the predicted binding site of the 3′-UTR-XIAP.

### 2.3. Increased Levels of miR-199a-5p Reduces XIAP Protein Expression

**Figure 3:**
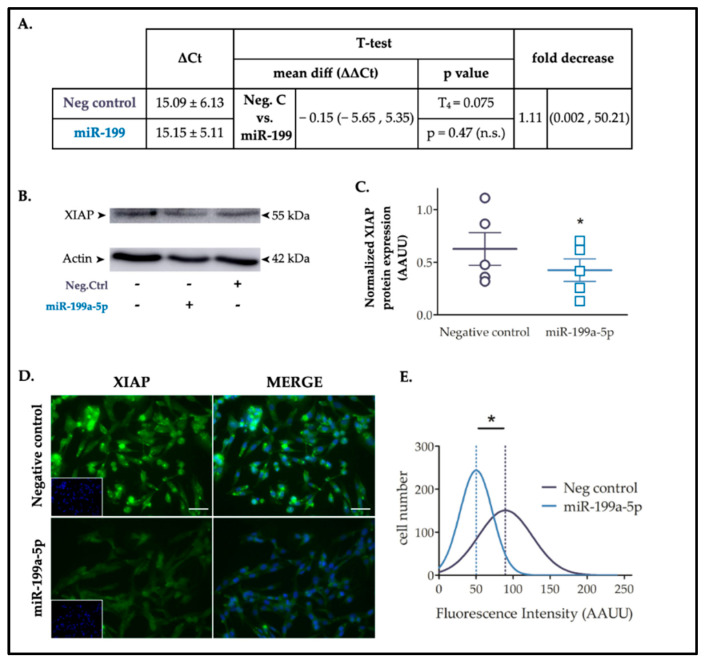
XIAP is a target of miR-199a-5p in C6 cells. (**A**) Statistical analysis of RT-qPCR results is provided in the table, employing a Student’s T test (n.s. = non significative). (**B**) Representative immunoblot of the expression levels of XIAP in protein samples extracted from C6 cells 24 h after transfection with either miR-199a-5p or negative control mimics. (**C**) Dot plot summary of the band densitometry from immunoblot analysis. Data were normalized by ß-actin levels for each sample. * *p*-value < 0.05 (paired *t*-test; n = 5 independent cell culture preparations). Lines represent mean ± SEM of n = 5 independent experiments. (**D**) Immunofluorescence assay of XIAP expression in non-transfected control, and negative control or miR-199a-5p mimic transfected C6 cells, labeled with a specific XIAP antibody (green) and DAPI (nuclei staining, blue). Bar scale = 100 μm. (**E**) Gaussian distribution graph of fluorescence intensity of XIAP staining in approximately 1500 cells per condition of C6 cells transfected with negative control (black line) or miR-199a-5p mimics (blue line). * *p*-value < 0.05 (Student’s *t*-test; n = 3 independent experiments with 5 images per experiment, analyzing approximately 300 cells per image). Dotted lines represent the mean fluorescence value for each distribution.

To confirm the effect of miR-199a-5p on XIAP expression, rat C6 cells were transfected with either miR-199a-5p or negative control mimics for 24 h, and both mRNA and protein levels were evaluated using RT-qPCR and immunoblot assays, respectively. RT-qPCR analysis showed no statistically significant difference in XIAP mRNA levels (t_4_ = 0.074; *p*-value = 0.47 in paired *t*-test; n = 3) (Figure 3A). However, immunoblots showed that miR-199a-5p significantly downregulated the level of endogenous XIAP protein (33 ± 4.25% reduction; t_4_ = 3.296 in a paired *t*-test; *p*-value = 0.03; n = 5) (Figure 3B,C). Consistently, XIAP immunofluorescence experiments revealed a similar trend after cell transfection with the miR-199a-5p mimic (Figure 3D). Analysis of endogenous XIAP fluorescence staining intensity per cell in C6 cell cultures transfected with miR-199a-5p or negative control mimics for 24 h revealed a significant reduction in mean fluorescence after miR-199a-5p transfection compared to negative control mimic-transfected cells (Gaussian distribution mean ± SD: Negative control 89.55 ± 36.2; miR-199a-5p mimic 50.25 ± 21.81; t_2_ = 3.327, *p*-value = 0.039 in *t*-test, n = 3; distribution values from approximately 3000 cells per condition) (Figure 3E). Taken together, these data suggest that overexpression of miR-199a-5p mediates a reduction in endogenous XIAP protein levels through translational repression without mRNA degradation.

### 2.4. Changes in miR-199a-5p and XIAP Expression After SCI

**Figure 4:**
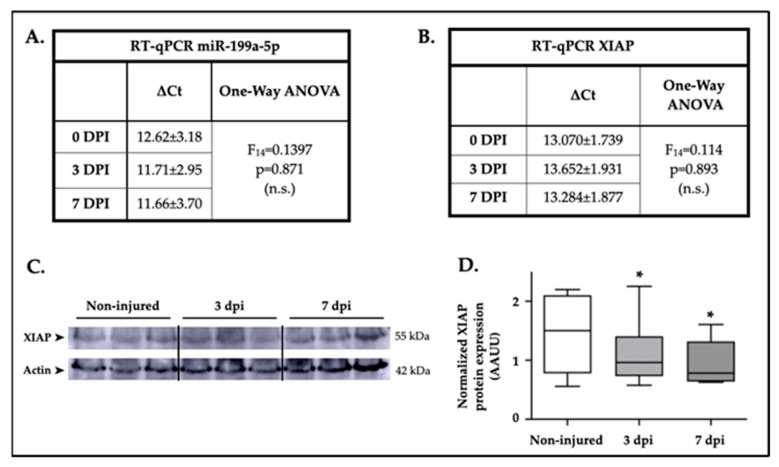
XIAP expression in the spinal cord was reduced after SCI. (**A**) Summary of RT-qPCR data showing miR-199a-5p gene expression levels in spinal cord samples at non-injured (0 dpi), 3 and 7 dpi. Results are based on data from five animals per group with 3 technical replicates each. Statistical analysis of the effect of dpi on gene expression is provided using one-way ANOVA (n.s. = non significative). (**B**) Summary of RT-qPCR data illustrating XIAP gene expression levels in spinal cord samples at non-injured, 3, and 7 dpi. The table presents results from five animals per group, with six technical replicates each. Statistical analysis of the effect of dpi on gene expression was performed using one-way ANOVA (n.s. = non significative). (**C**) Representative immunoblot depicting XIAP and β-actin protein expression in rat spinal cord samples at non-injured, 3, and 7 dpi from eight different animal surgery batches. (**D**) The box-and-whisker plots show XIAP protein levels in non-injured (white box), 3 dpi (light gray), and 7 dpi (dark gray) samples. Densitometry measurements were normalized to ß-actin levels for each sample (* *p* < 0.05 in paired *t*-test; n = 8 independent samples for each condition). In the box plots, the upper and lower boundaries represent the 25% and 75% quartiles, respectively, the line within the box marks the mean, and the whiskers above and below the box indicate the maximum and minimum values.

High-throughput expression data from previously published SCI studies (2–4) showed that, among all miRNAs predicted to target XIAP, only miR-199a-5p expression is increased at 3 and 7 days post-injury (dpi) (Figure 1C). To confirm these changes in miR-199a-5p expression and explore its effects on XIAP expression, we performed RT-qPCR and immunoblot analyses in spinal cord samples from non-injured (n = 5 animals per group) and injured rats at 3 and 7 dpi (n = 5). RT-qPCR results show that the overall expression of miR-199a-5p did not change significantly after injury (one-way ANOVA, F_14_ = 0.1397, *p*-value = 0.871), although a trend toward increased expression was observed at 3 and 7 dpi (Figure 4A). Analysis of XIAP gene expression in the same samples showed that its levels remained largely unchanged following injury (one-way ANOVA, F_14_ = 0.114, *p* = 0.893; Figure 4B). Interestingly, XIAP gene expression showed a decrease at 3 dpi, exhibiting an opposite trend to miR-199a-5p expression. To assess whether the upregulation of miR-199a-5p after SCI contributes to the downregulation of XIAP, we performed a Pearson correlation analysis of ΔCt values between miR-199a-5p and XIAP. The analysis yielded a correlation coefficient of Rs = −0.658 with a statistically significant *p*-value of 0.0069. This result indicates an inverse relationship, suggesting that increased levels of miR-199a-5p are associated with decreased levels of XIAP.

According to the trend observed in XIAP mRNA levels, XIAP protein levels in the spinal cord after injury showed a similar pattern to its gene expression. SCI resulted in a significant reduction in endogenous XIAP levels at 3 dpi (18% ± 9.36, t_7_ = 1.98, *p* = 0.043, paired *t*-test, n = 8 independent experiments) and at 7 dpi (22.5% ± 13.75, t_7_ = 1.97, *p* = 0.044, paired *t*-test, n = 8 independent experiments) relative to non-injured animals. These findings indicate a potential regulatory interaction between XIAP and miR-199a-5p, suggesting that the upregulation of miR-199a-5p following SCI may contribute to the observed downregulation of XIAP.

### 2.5. miR-199a-5p Expression and Distribution in the Naïve Rat Spinal Cord

**Figure 5:**
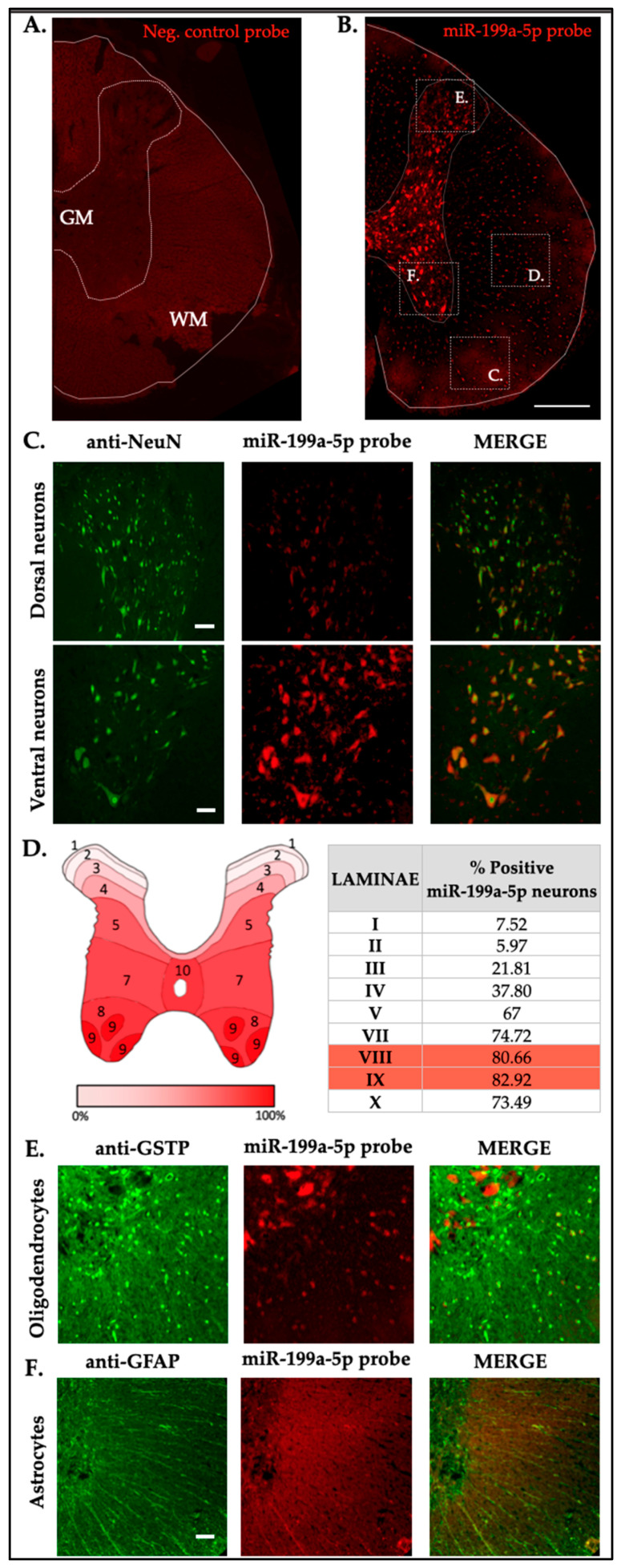
miR-199a-5p expression in neural cells of the undamaged rat spinal cord. (**A**) Representative image of a control spinal cord section from rats (n = 3) labeled with a “scrambled” probe. (**B**) Representative image of miR-199a-5p labeling in coronal sections of control spinal cords from rats (n = 3). (**C**) Co-staining of neurons using anti-NeuN (green, left), miR-199a-5p probe (red, middle), and their merged image (right), showing dorsal (upper panel) and ventral (lower panel) neurons. (**D**) Map of the Rexed laminae in the T9 spinal segment indicating the mean percentage of miR-199a-5p-positive neurons present in each lamina of the naïve spinal cord (4 sections from 3 individuals). Laminae with maximum percentage of miR-199a-5p positive neurons are showed in red. (**E**,**F**) Representative images showing co-labeling of oligodendrocytes (anti-GSTP) and astrocytes (anti-GFAP), both shown in green (left), with the miR-199a-5p probe (red, middle). Scale bar corresponds to 500 μm in panels (**A**,**B**), and to 50 μm in the neuron (**C**), oligodendrocyte (**E**), and astrocyte (**F**) panels.

The anatomical and cellular distribution of miR-199a-5p was examined using a combined fluorescence in situ hybridization (FISH) and immunofluorescence (IF) of cell-type-specific markers of main neural cells from the spinal cord, in non-injured T9 segment sections of rat spinal cords. miR-199a-5p labeling was heterogeneous across both white and gray matter tissues and neural cell types (Figure 5B).

In the gray matter, neurons labeled with anti-NeuN showed variable miR-199a-5p immunoreactivity (Figure 5C). Notably, intense staining was observed in large neurons of the ventral horn (Figure 5C, below), particularly in Rexed’s laminae VII, VIII, and IX, with staining intensity diminishing toward the dorsal horn (Figure 5C, above). Quantification confirmed heterogeneity in miR-199a-5p expression among Rexed’s laminae: only 10–20% of neurons in laminae I-III expressed miR-199a-5p, compared to 75–85% in the ventral laminae, predominantly in laminae VIII and IX (Figure 5D). On the other hand, we also found co-labelling of miR-199a-5p with oligodendrocytes, but in this case the intensity was discrete and variable throughout the area.

**Figure 6:**
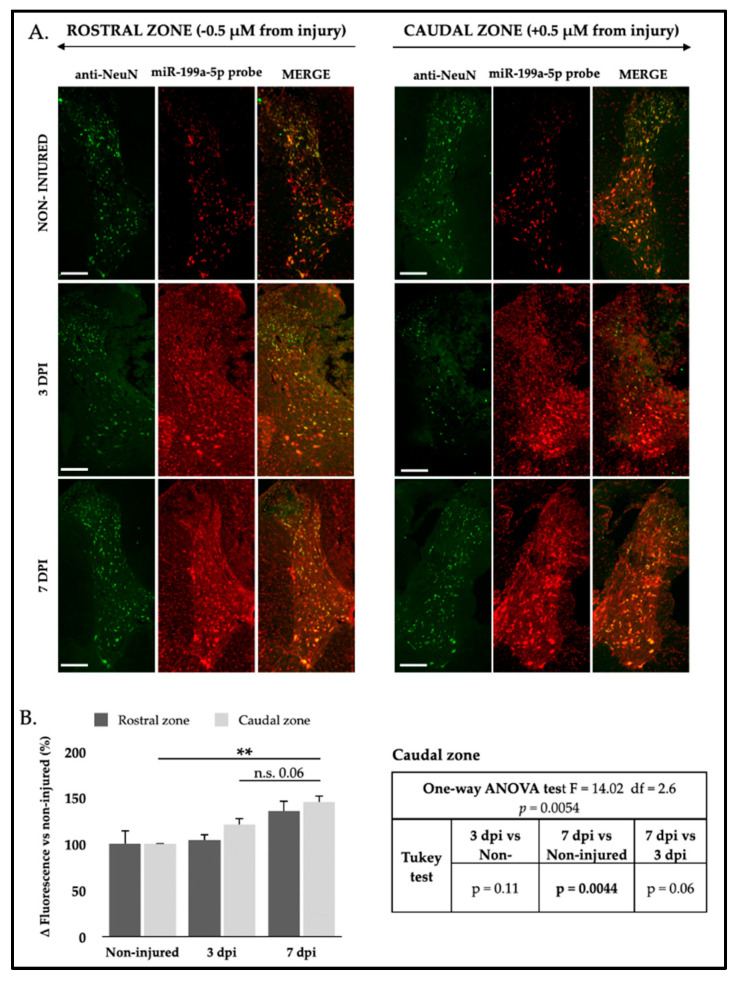
SCI significantly increases miR-199a-5p expression in neurons. Spatio-temporal distribution of miR-199a-5p expression in neurons after SCI. (**A**) Representative FISH-IF confocal images of rat spinal cords coronal sections of non-injured (upper row), 3 dpi (middle row), and 7 dpi (lower row), co-labeled with anti-NeuN antibody (green, left column) and miR-199a-5p probe (red, middle column) (n = 3 individuals per time). Sections are about 0.5 mm away from injury epicenter. Scale bar: 200 μm. (**B**) Analysis of miR-199a-5p staining intensity in rostral (dark gray bars) and caudal (light gray bars) spinal cord neurons in non-injured animals (0 dpi) and at 3 and 7 dpi. The graph represents the mean ± SD of sections from three animals per condition. Differences between conditions were analyzed using a one-way ANOVA test, followed by a Tukey’s post hoc test. ** denotes a significant difference (*p* < 0.01); n.s = non significative. For more detailed information on the analysis, please refer to the accompanying tables for the rostral zone (upper table) and the caudal zone (lower table).

In the white matter, miR-199a-5p staining was detected in oligodendrocytes (GSTP-positive cells) and showed different levels of labelling, with oligodendrocytes exhibiting both low and high levels of miR-199a-5p intensity (Figure 5D and Figure 6). However, the naive samples’ fluorescent labeling in oligodendrocytes was more subtle or even absent when compared to the neuronal labeling (Figure 5E). No staining was detectable among astrocytes (GFAP-positive cells; Figure 5F).

### 2.6. Changes in miR-199a-5p Expression After SCI

Using a combined FISH-IF assay, spinal cord sections from non-injured as well as 3 and 7 dpi animals were analyzed to assess potential changes in the expression of miR-199a-5p following spinal cord injury. Analyses focused on the penumbra zone surrounding the lesion (±0.5 mm) to avoid high autofluorescence in the injury epicenter. Compared to the gray matter non-injured spinal cord (Figure 6A), there were no significant changes in miR-199a-5p signal intensity at rostral sections (F_2,6_ = 2.33, *p* = 0.178; Figure 6B). However, a gradual significant increase in miR-199a-5p signal intensity was observed in caudal neurons at 3 and 7 dpi (F_2,6_ = 14.02, *p* = 0.0054; Figure 6B). Similarly to the analysis performed with neurons, changes in miR-199a-5p expression after SCI were examined in the white matter. Astrocytes were excluded from this study due to the absence of co-labeling with the miR-199a-5p-specific probe at both 3 and 7 dpi, indicating that these cells do not express miR-199a-5p under the examined conditions. This lack of co-localization is illustrated in Supplementary Figure S2, supporting their omission from further analysis.

**Figure 7:**
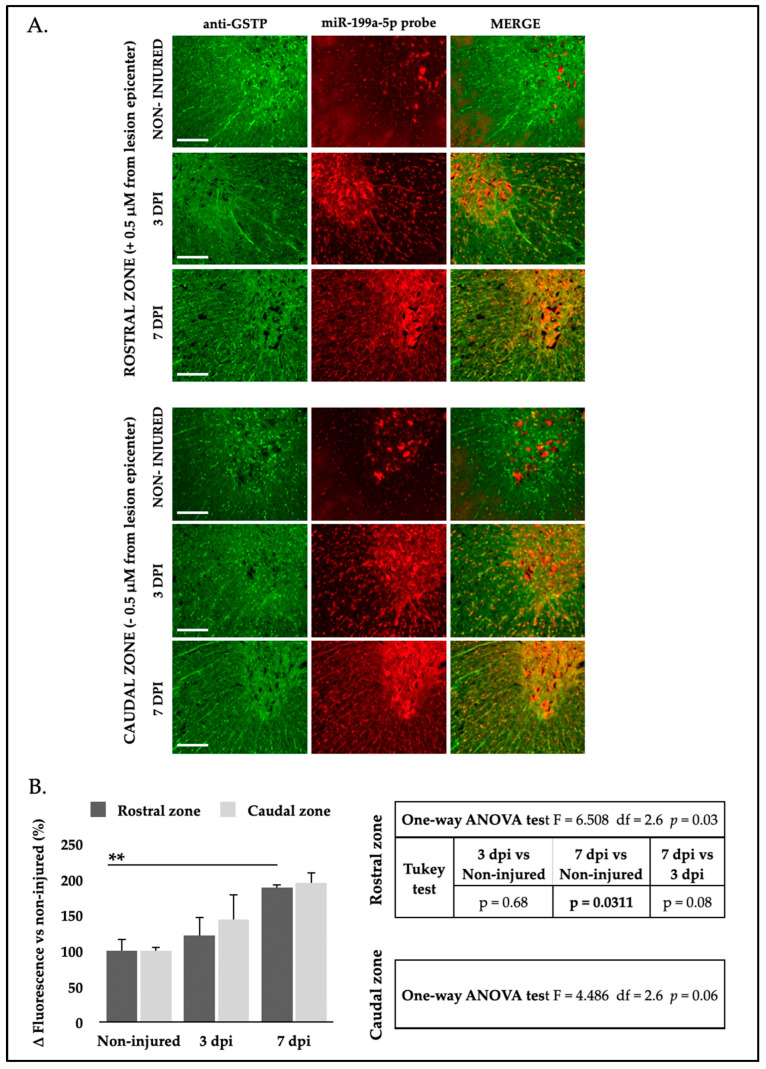
SCI induces a significant upregulation of miR-199a-5p expression in oligodendrocytes. (**A**) Representative high-resolution confocal FISH-IF images of coronal sections showing both gray and white matter areas from uninjured (top row), 3 dpi (middle row), and 7 dpi (bottom row) rat spinal cords, co-labeled with anti-GSTP antibody (green, left column) and miR-199a-5p probe (red, middle column), displaying rostral (top panel) and caudal (bottom panel) regions (n = 3 animals per time point). Scale bar: 200 μm. (**B**) Analysis of miR-199a-5p staining intensity in rostral (dark gray bars) and caudal (light gray bars) spinal cord oligodendrocytes in non-injured animals and at 3 and 7 dpi. The graph represents the mean ± SD of sections from three animals per condition. Differences between conditions were analyzed using a one-way ANOVA test, followed by a Tukey’s post hoc test. ** denotes a significant difference (*p* < 0.01) relative to non-injured condition. For more detailed information on the analysis, please refer to the accompanying tables for the rostral zone (upper table) and the caudal zone (lower table).

Regarding oligodendrocytes, analysis of FISH-IF images revealed a significant increase in miR-199a-5p signal intensity in these cells at 3 and 7 dpi. This increase was observed in oligodendrocytes within the white matter and in the neuropil of the gray matter, both rostral and caudal to the lesion (Figure 7A). Specifically, one-way ANOVA showed significant intensity changes in the rostral zone (F_2,6_ = 6.508, *p* = 0.031) peaking at 7 dpi (Tukey test 7 dpi vs. non-injured *p* = 0.031; Figure 7B) and a trend toward significance in the caudal zone at 7 dpi (F_2,6_ = 4.48, *p* = 0.06; Tukey test *p* = 0.051; Figure 7B). Altogether, histological findings indicate that injury significantly increases miR-199a-5p expression in neurons and oligodendrocytes of the damaged spinal cord with a marked increase observed at 7 dpi in the penumbra zone.

### 2.7. XIAP Expression and Distribution in the Naïve Rat Spinal Cord

**Figure 8:**
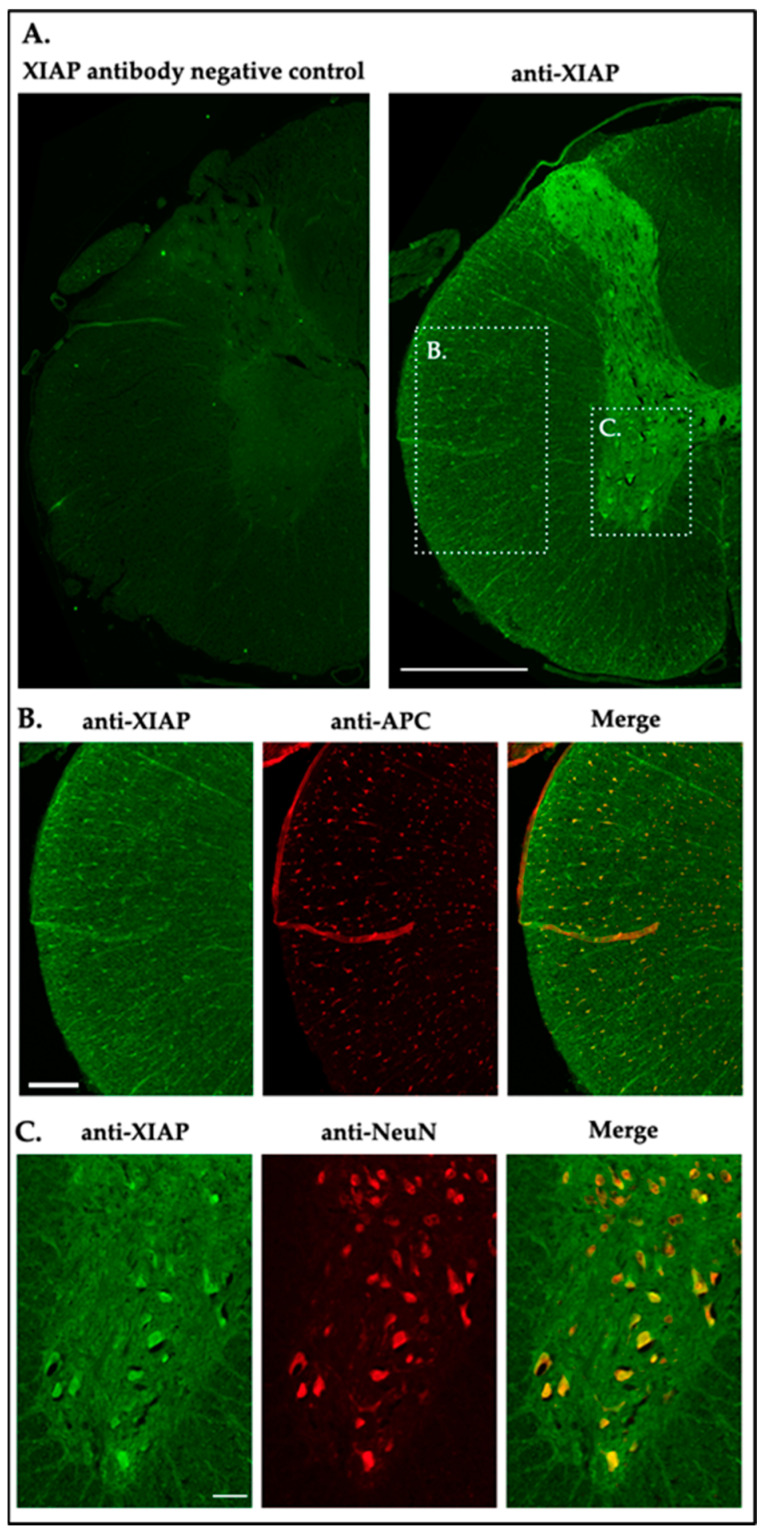
XIAP expression in neural cells of the undamaged rat spinal cord. (**A**) Image of negative control in absence of anti-XIAP antibody on a coronal section of non-injured rat spinal cord (left) and XIAP staining in coronal sections of control spinal cords from non-injured rats (right; scale bar: 200 μm; n = 3). (**B**,**C**) Confocal images of the different areas indicated in ((**A**), white dotted line) showing XIAP expression (green, left) and co-expression with cellular markers (red, right) for oligodendrocytes (anti-APC; scale bar: 200 μm; middle panel) (**B**), and ventral neurons (anti-NeuN; scale bar: 50 μm lower panel) (**C**).

IF was employed to assess the anatomical and cellular distribution of XIAP in non-injured rat spinal cords (Figure 8A). Similar to miR-199a-5p, XIAP labeling was heterogeneous across white (Figure 8B) and gray (Figure 8C) matters. XIAP was primarily detected in oligodendrocytes, showing varying levels of intensity (Figure 8B and see scatter plot in Figure 12B), while no immunoreactivity was observed in astrocytes using the astrocyte marker anti-GFAP. In the gray matter, neurons labeled with anti-NeuN displayed variable XIAP intensity, with diverse levels of staining in ventral horn neurons and weak signals in the dorsal horn (Figure 8C and see scatter plot in Figure 11B).

In summary, XIAP expression demonstrated a heterogeneous distribution in both white and gray matters. These findings suggest cell-type-specific XIAP expression patterns, with a stronger association in oligodendrocytes and ventral neurons.

### 2.8. XIAP Expression Changes After SCI

**Figure 9:**
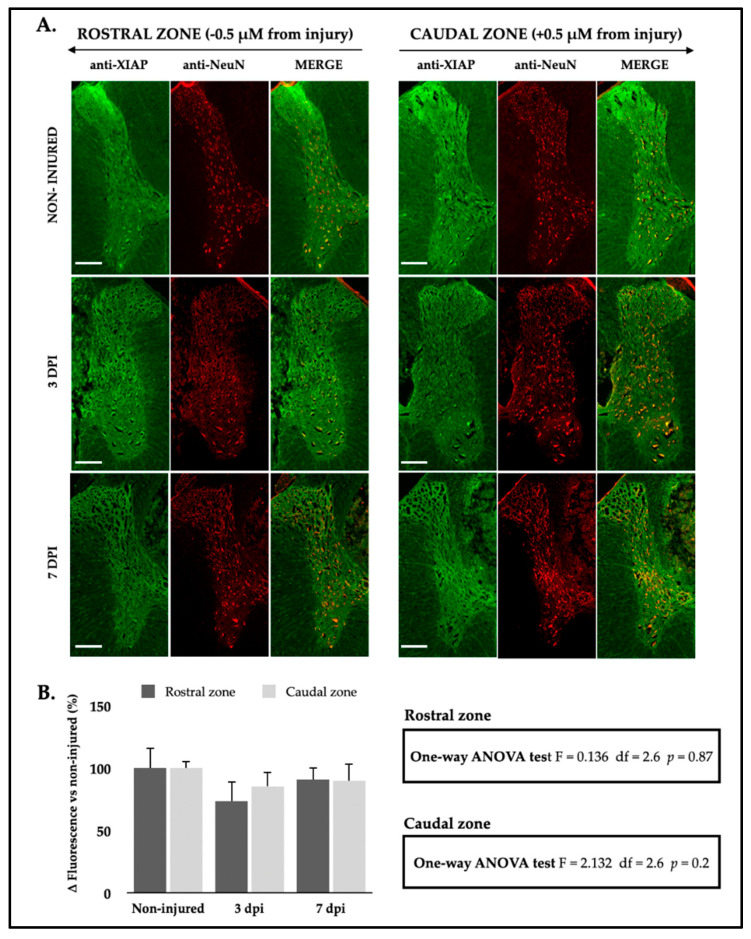
XIAP expression shows a tendency to decrease in neurons following SCI. (**A**) Representative IF confocal images of rat spinal cords hemi-coronal sections of non-injured (upper row), 3 dpi (middle row), and 7 dpi (lower row), co-labeled with anti-XIAP antibody (green, left column) and anti-NeuN antibody (red, middle column) (n *=* 3 individuals per time). Sections are about 0.5 mm away from injury epicenter. Scale bar: 200 μm. (**B**) Analysis of XIAP staining intensity in rostral (dark gray bars) and caudal (light gray bars) spinal cord neurons in non-injured animals (0 dpi) and at 3 and 7 dpi. The graph represents the mean ± SD of sections from three animals per condition. Differences between conditions were analyzed using a one-way ANOVA test. For more detailed information on the analysis, please refer to the accompanying tables for the rostral zone (upper table) and the caudal zone (lower table).

Changes in XIAP expression in the gray matter after SCI were analyzed using IF in rostral and caudal sections of rat spinal cords relative to the injury epicenter at 3 and 7 dpi. Interestingly, and contrary to the pattern observed with miR-199a-5p in neurons (Figure 9A), the intensity of XIAP in neurons appears to decrease in regions of the gray matter after 3 and 7 dpi. Although not statistically significant, the analysis indicated a decrease in XIAP fluorescence signal in neurons, particularly at 3 dpi in the rostral zone. This reduction was followed by a modest recovery in signal intensity by 7 dpi, to the baseline values observed in the uninjured condition (Figure 9B).

**Figure 10:**
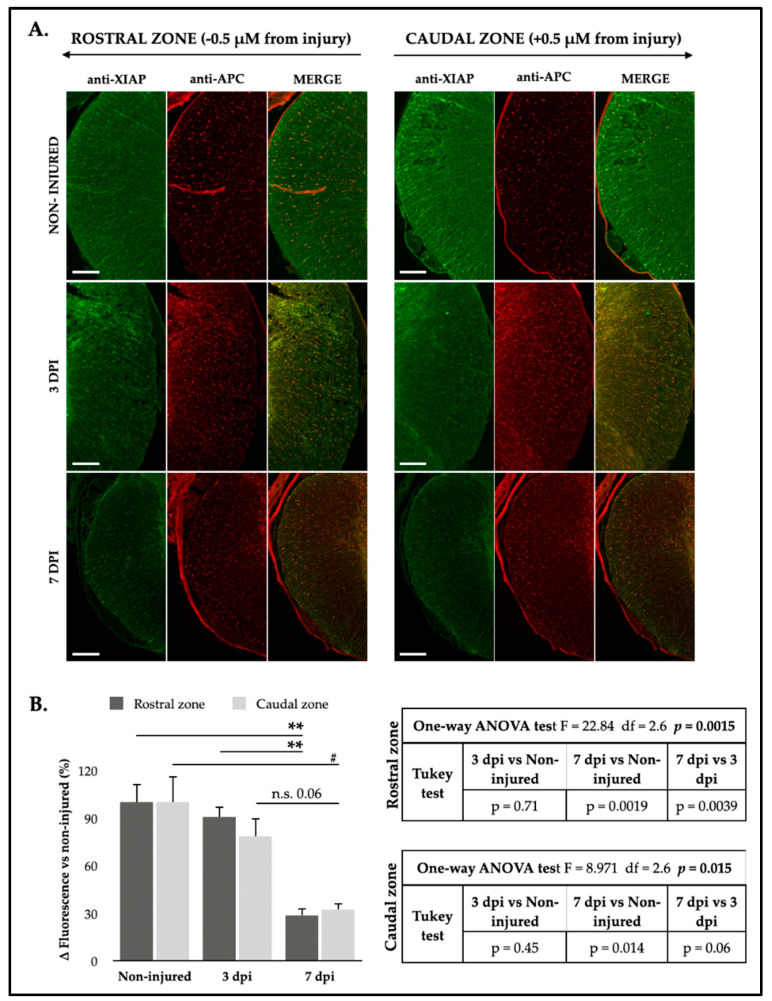
Spinal cord injury causes a significant decrease in XIAP in oligodendrocytes. (**A**) Representative IF confocal images of rat spinal cords hemi-coronal sections of non-injured (upper row), 3 dpi (middle row), and 7 dpi (lower row), co-labeled with anti-XIAP antibody (green, left column) and anti-APC antibody (red, middle column) (*n* = 3 individuals per time). Sections are about 0.5 mm away from injury epicenter. Scale bar: 200 μm. (**B**) Analysis of XIAP staining intensity in rostral (dark gray bars) and caudal (light gray bars) spinal cord neurons in non-injured animals (0 dpi) and at 3 and 7 dpi. The graph represents the mean ± SD of sections from three animals per condition. Differences between conditions were analyzed using a one-way ANOVA test, followed by a Tukey’s post hoc test. ** denotes a significant difference (*p* < 0.01), # denotes a significant difference (*p* < 0.05), and n.s. denotes non significative respectively, relative to non-injured condition. For more detailed information on the analysis, please refer to the accompanying tables for the rostral zone (upper table) and the caudal zone (lower table).

XIAP labeling was heterogeneous in the white matter after SCI, similar to the pattern observed in non-injured tissue, but conversely, it showed a decreasing trend following injury (Figure 10A). Analysis of IF images revealed that SCI significantly reduced XIAP fluorescence intensity in penumbra oligodendrocytes, with a marked decrease at 7 dpi (rostral zone: one-way ANOVA, F_2,6_ = 22.84, *p* = 0.0015; caudal zone: one-way ANOVA, F_2,6_ = 8.971, *p* = 0.015; Figure 10B). These results indicate that SCI leads to a decrease in XIAP expression mainly in oligodendrocytes and slight in neurons within the penumbra zone, particularly at 7 dpi.

### 2.9. XIAP-miR-199a-5p Expression Changes After SCI

**Figure 11:**
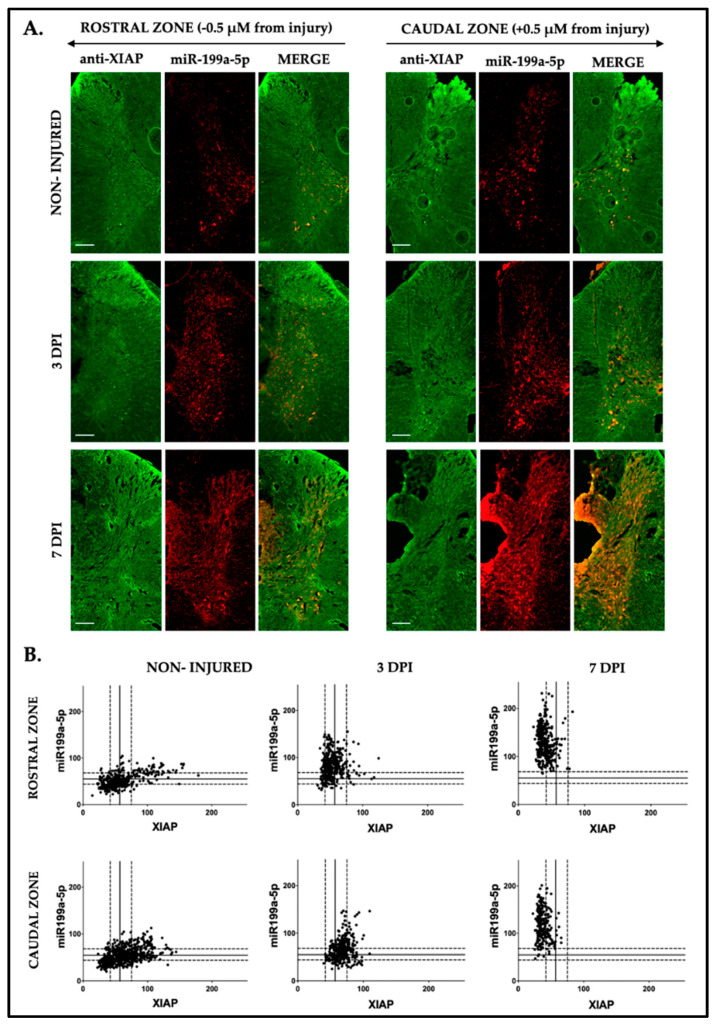
Dynamic and dependent relationship between miR-199a-5p and XIAP in neuronal cells following spinal cord injury. (**A**) Representative FISH-IF confocal images of rat spinal cords hemi-coronal sections of non-injured (upper row), 3 dpi (middle row), and 7 dpi (lower row), co-labeled with anti-XIAP antibody (in green, left column) and miR-199a-5p probe (in red, middle column) (n = 3 individuals per time). Sections are about 0.5 mm away from injury epicenter. Scale bar: 200 μm. (**B**) Representative scatter plot images are shown from rostral (upper panel) and caudal zones (lower panel), with each point corresponding to the intensity values of miR-199a-5p (Y-axis) and XIAP (X-axis) per cell. Solid lines indicate the mean fluorescence intensity, while dotted lines represent the ± SD from the mean (n = 3 individuals per time).

Co-expression changes in XIAP and miR-199a-5p in the gray matter were analyzed using IF and FISH analysis in rostral and caudal sections of rat spinal cords relative to the injury epicenter at 3 and 7 dpi (Figure 11A). The observed results are consistent with those reported in previous sections, showing an increase in miR-199a-5p labeling over time after SCI, accompanied by a parallel reduction in XIAP expression within the same cells. Following SCI, dorsal neurons exhibited a marked increase in miR-199a-5p signaling, alongside a reduction in XIAP labeling. This labeling pattern is even more pronounced in the ventral region, where it is predominantly focused in motoneurons. These changes are effectively illustrated in the scatter plot of miR-199a-5p/XIAP expression. In uninjured samples (Figure 11B), XIAP and miR-199 expression values exhibit a dispersed and heterogeneous plot pattern. However, as SCI progresses, this pattern shifts, with XIAP labeling becoming more concentrated and miR-199a-5p labeling expression becoming increasingly scattered and elevated.

**Figure 12:**
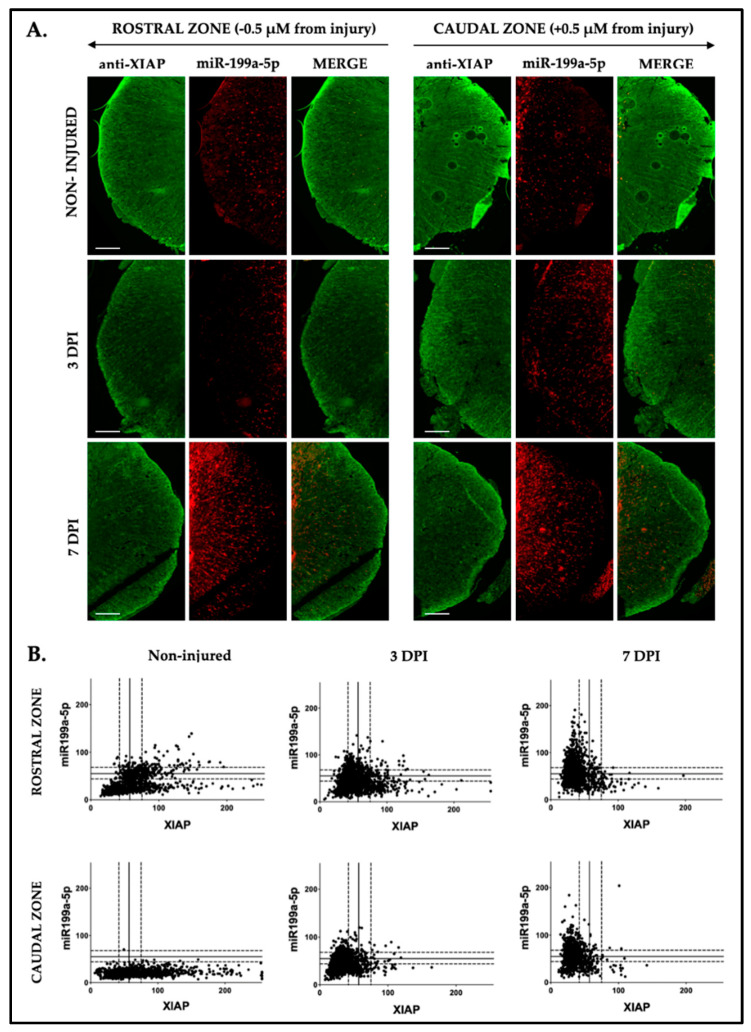
Dynamic and dependent relationship between miR-199a-5p and XIAP in oligodendrocytes following spinal cord injury. (**A**) Representative FISH-IF confocal images of rat spinal cords hemi-coronal sections of non-injured (upper row), 3 dpi (middle row), and 7 dpi (lower row), co-labeled with anti-XIAP antibody (in green, left column) and miR-199a-5p probe (in red, middle column) (n = 3 individuals per time). Sections are separated about 0.5 mm from injury epicenter. Scale bar: 200 μm. (**B**) Representative scatter plot images are shown from rostral (upper panel) and caudal zones (lower panel), with each point corresponding to the intensity values of miR-199a-5p (Y-axis) and XIAP (X-axis) per cell. Solid lines indicate the mean fluorescence intensity, while dotted lines represent the ±SD from the mean (n = 3 individuals per time).

In the white matter, this plot pattern is more pronounced, with increasing miR199a-5p labeling levels observed at 3 and 7 dpi with a corresponding reduction in XIAP labeling expression (Figure 12A). As Figure 12B shows, over time following the injury, XIAP expression decreases below its median expression labeling level, while miR-199a-5p expression rises above its median both in rostral and caudal sections. Thus, SCI results in a time-dependent increase in miR-199a-5p expression and a decrease in XIAP expression. This shift is evident in neurons of the ventral sections and particularly pronounced in oligodendrocytes, with miR-199a-5p levels rising and XIAP levels notably declining at 3 and 7 days post-injury.

In summary, our analysis reveals a region-specific modulation of XIAP by miR-199a-5p following SCI in both white and gray matter. As injury progresses, miR-199a-5p expression rises, both in neurons and oligodendrocytes, while XIAP levels show an inverse reduction. Scatter plot analyses underscore a marked shift in expression dynamics, with miR-199a-5p displaying increased scatter and elevated levels, while XIAP becomes more tightly clustered and diminished. Taken together, these data support the idea that miR-199a-5p acts as a post-transcriptional regulator of XIAP, potentially exacerbating apoptotic susceptibility and cellular dysfunction following spinal cord injury.

## 3. Discussion

The main aim of the present work is to identify miRNAs with altered expression after SCI that potentially modulate XIAP expression, employing a bioinformatic approach and subsequently investigating this interaction in both an in vitro and in vivo SCI model. Our findings are novel in identifying miR-199a-5p as a specific regulator of XIAP in the context of SCI, suggesting its potential role in neural cell death regulation. Additionally, the spatial distribution analysis of miR-199a-5p and XIAP in the rat spinal cord highlighted the heterogeneous expression patterns in different neural cell types and regions, adding another layer of complexity to the regulation of apoptosis in SCI.

Apoptotic cell death is a major event affecting neural cells in the secondary injury driven by an imbalance of pro- and anti-apoptotic regulators [34]. Our previous studies and others showed a downregulation of the anti-apoptotic protein XIAP and an upregulation of the caspase activation in the first days after SCI, increasing the risk of apoptosis of neural cells [26,28,35,36]. MicroRNAs play a significant role among these apoptotic regulators, with the damaged spinal cord altering miRNA-mediated post-transcriptional regulation of key genes involved in the secondary cascade of events that lead to cell demise [6,33,37,38,39].

Our bioinformatic studies identified a total of six miRNA candidates as regulators of XIAP, among which the downregulated miR-340-5p and some miR-181 family members have been related to SCI [40,41,42]. On the other hand, the upregulation of the candidate miR-21a-5p may have a protective effect on neurons after SCI by the modulation of the PDCD4/caspase-3 pathway [43]. We mainly focused on miR-199a-5p specifically expressed in nerve tissues of human and mouse species [44,45,46,47,48] that become upregulated after SCI according to previous SCI high-throughput studies [6,32,33] and which provided the highest accessibility scores at the evaluation of the binding miR-199a-5p to the MREs in the sequence of the mRNA of XIAP.

Luciferase gene reporter assays confirmed the binding of miR-199a-5p to one specific site of XIAP 3′UTR mRNA. Furthermore, the effective regulation of endogenous XIAP expression by miR-199a-5p overexpression was demonstrated in cell culture, as evidenced by significant reductions in protein levels. However, while the administration of the miR-199a-5p mimic significantly reduced XIAP protein levels in cell cultures, it did not appear to affect XIAP transcript abundance. This result aligns with previous findings, such as those involving miR-24, which also did not mediate XIAP repression through mRNA degradation [49]. The silencing of XIAP via miRNA interaction with its 3′UTR can occur through mRNA degradation, translational repression, or an interplay of both mechanisms [50]. Although our in vitro data suggest that miR-199a-5p primarily exerts its inhibitory effect via translational repression rather than mRNA degradation, the possibility of other microRNA-mediated regulatory mechanisms contributing to XIAP expression cannot be ruled out.

Regulation of miR-199a-5p on XIAP expression may have a functional impact on the triggered processes of the secondary damage after SCI such as apoptotic cell death. Emerging evidence has shown that miR-199a-5p inhibition plays a neuroprotector role in several neurological disorders through regulating proteins involved in cell death or survival pathways. For instance, inhibition of miR-199a-5p has been shown to protect neurons against apoptosis and reactive oxygen species (ROS) generation by overexpressing its target protein Brg1 in a cerebral ischemia/reperfusion injury model [51]. Additionally, in rat models of cerebral ischemia, miR-199a-5p inhibition has induced ischemic tolerance by upregulating Sirt1 [27]. Similarly, in ischemic stroke models, downregulation of miR-199a-5p has been demonstrated to protect neurons against apoptosis and enhance cell viability through the CAV-1/MEK/ERK axis [52], while also improving cognitive function and reducing neuronal apoptosis in the hippocampus via activation of the AKT signaling pathway [53]. In the context of SCI, the neuroprotective effects of miR-199a-5p inhibition have also been reported. Gao and colleagues reported that downregulation of miR-199a-5p might mediate the therapeutic benefits of olfactory ensheathing cells in SCI rats [54]. Moreover, they found that the neurotoxicity elicited by miR-199a-5p overexpression in the spinal cord could be mitigated by using antagomiRs against miR-199a-5p, suggesting a potential therapeutic strategy for ameliorating SCI [53].

As previous studies have demonstrated the critical role of miRNAs as regulators of apoptotic pathways following neural injuries [5,55], our study further supports this by validating the regulation of XIAP expression by miR-199a-5p. This suggests that miR-199a-5p may play a significant role in the secondary damage processes following SCI, particularly in promoting apoptotic cell death. Notably, we observed a miR-199a-5p overexpression at 7 dpi, contrasting with the reduction in endogenous XIAP expression previously reported [26,28,35,36] and confirmed at the protein level in our study. Our correlation analysis of miR-199a-5p and XIAP gene expression yielded an inverse relationship, suggesting that increased levels of miR-199a-5p are associated with decreased XIAP expression. This inverse relationship was consistent with the pattern observed in XIAP protein levels, where SCI induced a significant reduction in XIAP expression at both 3 dpi and 7 dpi compared to non-injured controls. These findings collectively support a potential regulatory interaction between XIAP and miR-199a-5p, implicating miR-199a-5p overexpression as a contributing factor to the downregulation of XIAP following SCI.

However, these data results from a combination of injured, penumbrae, and spared spinal cord tissue, involving heterogeneous mixtures of spinal and infiltrating cells. This heterogeneity may mask the specific cell origins of the observed gene expression changes and potentially overlook significant gene expression alterations in less-represented cell types [56]. Furthermore, miR-199a-5p expression changes in the damaged spinal cord may vary depending on the injury model used, as has been described for other miRNAs following SCI (e.g., miR-21 upregulation [57] versus downregulation [58]). For example, upregulation of miR-199a-5p has been observed in contusive injury models (Figure 4A and [54]) and neurotoxicity-induced models [59,60], while downregulation has been reported in an ischemia-reperfusion model [61]. Thus, understanding the origins of these differences in miRNA expression could be key for developing targeted RNA-based therapeutic applications in the future.

Since these observed changes suggest that the upregulation of miR-199a-5p in the spinal cord after injury may contribute to the downregulation of XIAP, we performed a cellular-level analysis of miR-199a-5p and XIAP distribution in the naïve spinal cord, as well as their expression changes following SCI.

In the non-injured rat spinal cord, miR-199a-5p expression exhibited a heterogeneous distribution across both white and gray matter, with notable intensity in large neurons of the ventral horn, particularly in Rexed’s laminae VIII and IX. In the white matter, miR-199a-5p was present in oligodendrocytes, though with varying levels of miR-199a-5p staining intensity. Although miR-199a has been reported as inhibitor of astrocyte activation in brain injury [62], our results in traumatic spinal cord injury model in adult rat indicates a lack of miR-199a and GFAP co-staining in FISH/IF assay. Following SCI, there was a significant increase in miR-199a-5p expression in both neurons and oligodendrocytes, particularly at 7 dpi, with the upregulation being most pronounced in the caudal region at 7 dpi. These findings agree with an uneven miRNA expression pattern in the naive spinal cord as well as the expression pattern changes described following SCI of the neuronal-specific kind of the miR-138-5p, miR-124, or the miR21 [14,63,64]. Conversely, previous studies have reported alterations in XIAP expression following SCI; however, these changes have not been observed at the single-cell level or anatomically regionalized within specific areas [26,28,35,36]. Our findings show that XIAP protein exhibited a non-uniform distribution across white and gray matter regions of non-injured rat spinal cord, with a more pronounced presence in oligodendrocytes and ventral horn neurons. Following SCI, the findings suggest a reduction in XIAP expression, predominantly in oligodendrocytes and slight in neurons located within the penumbra region at 7 dpi. These findings suggest that SCI induces a time-dependent increase in miR-199a-5p expression, contributing to a region-specific modulation of XIAP, which may exacerbate apoptotic cell death in the injured spinal cord.

Despite the thorough experimental validation of miR-199a-5p’s role in regulating XIAP, including analyses at the bioinformatic, in vitro, and in vivo levels, several limitations remain. Notably, our study concentrated on a single miRNA–XIAP interaction and did not investigate potential compensatory mechanisms involving other miRNAs or regulatory pathways. XIAP expression is regulated by diverse post-transcriptional mechanisms that influence its stability, degradation, and localization. For instance, RNA-binding proteins like HuR stabilize XIAP mRNA [65], while ubiquitin ligases, such as Smac/DIABLO and HTr2A/OMI, promote its degradation [66,67]. Post-translational modifications, including phosphorylation and SUMOylation, further influence XIAP stability, and other competing endogenous RNAs (ceRNAs) may modulate XIAP by sequestering miRNAs that target it, effectively reducing miRNA-mediated repression [68,69,70]. Moreover, caspase-3, caspase-7, and the protease calpain can cleave XIAP, decreasing its stability in a feedback loop that promotes apoptosis and cellular balance [19,71,72]. Our research was limited to effects of miR-199a-5p and XIAP regulation at the acute phase following SCI. Our study focused on miR-199a-5p’s effect on XIAP in the acute phase of SCI, but future work should examine these dynamics across different SCI models and time points. Investigating additional apoptotic regulators alongside miR-199a-5p could further clarify SCI apoptotic pathways, highlighting the therapeutic potential of miR-199a-5p inhibition or XIAP overexpression. Such approaches may help reduce cell demise and improve functional recovery following spinal cord injury.

## 4. Materials and Methods

### 4.1. Bioinformatics and Data Mining

We used an in silico screening approach, combining computational tools that employ existing databases and prediction algorithms, and data mining for gene expression data analysis, to predict miRNAs (miRNAs) candidates with microRNA response elements (MREs) in the 5′-UTR and 3′-UTR and the coding region of the rat mRNA of XIAP and that can be used as therapeutic tools reducing SCI-induced cell death. We used the following four prediction tools: microRNA, Target Scan (http://www.targetscan.org; last accessed 14 Febreruary 2020), miRMap (https://mirmap.ezlab.org/; last accessed 14 Febreruary 2020), and miRWalk (http://mirwalk.umm.uni-heidelberg.de/; last accessed 14 Febreruary 2020). Already validated miRNA–target interactions were explored using miRTarBase 6.0 database (https://mirtarbase.cuhk.edu.cn/~miRTarBase/miRTarBase_2019/php/index.php; last accessed 14 March 2020). We studied miR-199a-5p MRE accessibility of the mRNA of XIAP using (i) mFold tool (http://www.unafold.org/; last accessed 15 April 2020) [73] to calculate the free energy of the binding site in comparison with the free energy of the 100 nucleotides flanking the 3′UTR at both 5′ and 3′ sides, and (ii) miRMap program to calculate the minimal free energy as a measurement of accessibility, computing stability degree of miRNA–mRNA duplexes.

### 4.2. Spinal Cord Injury Model

In vivo procedures were performed in female Wistar rats (of 200 g of weight (12–14 weeks of age; RRID:RGD_13508588)). Animals were bred at the Animal Facility of the Research Unit and housed in plastic cages in a temperature and humidity-controlled room maintained on a 12:12 h reverse light/dark cycle with free access to food and water. SCI surgery followed the methodology described by Yunta and col [6]. Briefly, following thoracic vertebra 8 (T8) laminectomy, rats were injured by a 200 KDyne contusion (IHSpinal Cord Impactor device from Precision System & Instrumentation; Lexington, KY, USA). After surgery, animals were maintained by daily manual bladder expression and by administration of the analgesic Buprenorphine (0.03 mg/Kg Buprex; Reckitt Benckiser Pharmaceuticals Limited; Richmond, VA, USA), and the antibiotic enrofloxazine (0.4 mg/Kg Baytril; Bayer AG; Leverkusen, Germany) up to 2 days after injury. Hind limb paralysis after injury was confirmed 2 days after the surgery using the Basso, Beattie, and Bresnahan 21-point locomotor score for rat models of SCI (BBB; [74]). We used a BBB scale value of 7 at 2 days post-injury (dpi) as the upper limit to include the animals in the gene expression analyses. We distributed animals in three experimental groups, 3 and 7 dpi and non-injured control, each comprising three individuals. Animals were randomly distributed in the experimental groups following this procedure: each animal received an arbitrary number and was allocated to one of the experimental groups or a reserve group using a random sequence generated with https://www.random.org/. The first three animals in the sequence were allocated to the control group, the second three to the 3 dpi group, the following three individuals formed the 7 dpi group, and the remaining individuals were ordered according to the random sequence to be employed as reserve individuals. The first reserve subject replaced one individual from the 7 dpi group that was excluded due to a suboptimal contusion, as evidenced by an excessive locomotor recovery score above 7 on the BBB scale at 2 dpi, under the established exclusion criteria. Animals were subjected to surgeries on different days so that all could be sampled on the same day. All manipulations and treatments were carried out in full accordance with the guidelines on the care and management of animals established by the European Union (directive 86/609/CEE), the guidelines on the use of animals for Neuroscience Research of the Society for Neuroscience, the NIH guide for the care and use of laboratory animals, and the normative R.D. 1201/2005 10-10 from the Spanish Ministry of the Environment and the Agriculture Council of the Castilla-La Mancha animal ethics committees. All procedures were approved by the Animal Care and Use Committee of Hospital Nacional de Paraplejicos (153BCEEA/2016). All efforts were made to minimize suffering as well as the number of animals used.

### 4.3. Cell Culture

The C6 rat brain glioma cell line (cat#: CRL-2266, RRID:CVCL_0194, ATCC; Manassas, VA, USA) was grown in RPMI-1640 medium (Gibco, Life Technologies, Carlsband, CA, USA) supplemented with 10% fetal bovine serum (FBS; Gibco), 100 U/mL penicillin/streptomycin (Gibco) and 1× glutamine (Gibco). Cells were cultured in a humidified incubator at 37 °C in a controlled atmosphere containing 5% CO_2_. The specific culture plates and cell densities and counts used in each experimental setting are described in their corresponding methodological section.

### 4.4. Transfections

Transfection was carried out according to the recommended procedures by Dharmafect-4 reagent manufacturer (Dharmacon, Horizon Discovery; Waterbeach, UK). In brief, cell cultures were transfected for 24 h with either 50 nM of miR199a-5p mimic (miRBase accession number: MIMAT0000231; miRIDIAN hsa-miR-199a-5p mimic, Dharmacon cat#: C-300533-03-0002, mature sequence: 5′cccaguguucagacuccuguuc) or a negative control, the cel-miR-67-3p mimic from *C. elegans*, that has minimal sequence identity with any human, mouse, or rat miRNAs (miRBase accession number: MIMAT0000039; miRIDIAN miRNA mimic negative control #1, Dharmacon cat#: CN-001000-01-05; mature sequence: 5′-ucacaaccuccuagaaagaguaga).

### 4.5. RT-qPCR

To carry out RT-qPCR on spinal cord samples, animals were sacrificed by sodium pentobarbital overdose at the defined times (0 (non-injured), 3, or 7 dpi). To employ the same samples for qPCR and immunoblotting, we perfused the animal with 50–100 mL of PBS 1× + heparine (1 unit/mL; Chiesi España, Barcelona, Spain) (flux of 30 mL/min) to eliminate blood in the spinal cord. Then, one cm long spinal cord fragments centered in the injury area were extracted (approximately 70 mg), and manually homogenized in 300 µL of 25 mM HEPES, pH 7.5 (Merck; Darmstadt, Germany) supplemented with a protease inhibitor cocktail (Roche; Bassel, Switzerland) using RNAse free sterile pellet pestles (Fisher Scientific; Waltham, MA, USA). The so-obtained homogenate was then divided and processed independently for RNA and protein extraction. Samples were coded by a member of the laboratory that did not participate in the RT-qPCR so that all subsequent processes were blinded for the researchers in charge of analyzing the samples. C6 cells (10^6^ cells) were plated in 35 mm dishes. After reaching 80% confluence, cultures were transfected for 24 h with 50 nM of either miR-199a-5p or negative control mimics. Total RNA samples of spinal cords and C6 cultures were extracted using the Qiazol Lysis Reagent (Qiagen; Hilden, Germany) followed by purification using the miRNeasy Isolation Kit (Qiagen) according to manufacturer protocols. RNA content in each sample was determined using an ND 1000 spectrophotometer (NanoDrop Technologies Fisher Scientific). To determine miR-199a-5p expression, 10 ng of total RNA was reverse-transcribed and amplified using TaqMan miRNA gene expression-specific probe (TaqMan^®^ miRNA assay #000498, Applied Biosystems; Foster city, CA, USA) following the manufacturer ’s protocols. The U6 small nuclear RNA served as an internal control (U6 probe TaqMan^®^ miRNA assay #001973, Applied Biosystems). To evaluate mRNA of XIAP content, 1 μg of total RNA was treated with DNase I (Roche) for 30 min at 37 °C plus 3 min at 95 °C and then retrotranscribed by incubation with Moloney leukemia virus transcriptase (Invitrogen; Fisher Scientific) and Primer Random mix (Roche) for 60 min at 37 °C plus 3 min at 95 °C. The amplification reaction of both miRNAs and mRNA retrotranscribed samples was performed following the ∆∆Ct routine (see details in: https://assets.thermofisher.com/TFS-Assets/LSG/manuals/4364016.pdf; accessed on 15 November 2024) in a TaqMan 7900HT Fast Real-Time PCR System (Applied Biosystems) using the TaqMan Universal PCR Master Mix, no AmpErase UNG (Fisher Scientific) together with commercial specific FAM-MBG conjugated probe for XIAP mRNA (Life Technologies; cat#4331182; Rn01457299_m1) and miR-199a-5p miRNA (gene expression-specific probe, TaqMan^®^ MicroRNA assay cat#000498). 18S ribosomal RNA (for mRNA; Life Technologies, cat#4333760; Hs99999901_s1) and U6 RNA (for miRNA; TaqMan^®^ MicroRNA assay cat#001973, Applied Biosystems) served as internal control. The reactions were programmed in the 9600 emulation mode; that is, first 10 min at 95 °C, followed by 40 cycles of a two-step amplification run, comprising 15 s at 95 °C, plus 1 min at 60 °C using ABI Prism 7900 fast thermocycler (Applied Biosystems).

Both miRNA and mRNA data were analyzed following the methods from Livak and Schmittgen [75]. Briefly, we determined the difference (∆Ct) between the target mRNA’s or miRNA’s cycle threshold and their respective endogenous loading controls and its associated variance following the standard propagation of error method from Headrick and col [76]. Then, we compared the ∆Ct value from the miR-199a-5p mimic condition with the ∆Ct from the negative control sequence to calculate the ∆∆Ct and the correspondent fold increase (2^−∆∆Ct^), also indicating the 95% confidence interval. Statistical analysis was performed using a one-way ANOVA with Tukey post hoc test.

### 4.6. Dual-Luciferase Reporter Gene Construction and 3′UTR Luciferase Reporter Assays

The wild-type (wt) 3′UTR sequence of rat XIAP mRNA (XIAP 3′UTR-wt; NCBI Reference Sequence: NM_022231.2) containing the predicted binding site for rno-miR199a-5p (nt 2255-2262) was obtained from total rat brain DNA extract by amplification by PCR (Table 1). The amplified sequence was subcloned into the T vector plasmid (pGEM-T-easy, Promega) and a pBKS vector (pBluescript, Stratagene; San Diego, CA, USA). The 3′UTR sequence was validated by DNA sequencing (T7p and SP6). After amplification by transformation in E.coli super-competent cells (Thermo Scientific), the XIAP 3′UTR-wt sequence was inserted into the pmiRGLO Dual-luciferase miRNA Target Expression Vector (Promega, Fitchburg, WY, USA; a scheme on the reporter construct is available at (http://www.addgene.org/vector-database/8236/; accessed on 15 November 2024) between the SacI and XbaI restriction sites (pmiRGLO^XIAP^) using the FastDigest restriction enzymes (Thermo Scientific). Following a similar strategy as the QuikChange Site-Directed Mutagenesis (Thermo Scientific), a 3′UTR point mutant sequence (XIAP 3′UTR-mut) was generated by PCR using the XIAP 3′UTR-mut primers (Table 1) and the PfuI polymerase (Thermo Scientific), and the pBKS plasmid with the XIAP 3′UTR-wt subcloned serving as template, after DpnI endonuclease restriction digestion (FastDigest, Thermo Scientific). After amplification by transformation in E.coli super-competent cells, the XIAP-3′UTR-mut fragment was inserted into pmiRGLO between the SacI and SalI restriction sites (pmiR-GLO^XIAP-mut^). Finally, we confirmed the sequence of both pmiRGLO XIAP 3′UTR constructs by DNA sequencing using a specific forward 3′ end luciferase primer.

C6 cells were grown at 80% confluence (10^4^ cells per well in 96-well plate) and co-transfected with 50 nM of miR-199a-5p or negative control (cel-miR-67) mimics and 2 μg/mL of pmiRGLO^XIAP^ or pmiR-GLO^XIAP-mut^, using the DharmaFECT Duo Transfection Reagent (Dharmacon; Horizon Discovery). After 24 h, we measured the firefly luciferase to renilla luciferase light emission ratio according to the manufacturer’s protocol (Dual-Luciferase Reporter Assay System, Promega) using a spectrophotometer plate reader (Infinite M200, Tecan Group LTD; Mannendorf, Switzerland). Firefly emission data were normalized to renilla load control levels and expressed as the firefly/renilla ratio.

### 4.7. Immunoblotting Assay

For the analysis of protein expression in rat samples, 100 µL of homogenate of spinal cord, obtained as described in Section 4.5, was diluted in RIPA lysis buffer (Sigma; St Louis, MO, USA) supplemented with Complete EDTA-free protease inhibitor cocktail (Merck). For in vitro analyses, C6 cells were cultured in a 6-well plate (2.5 × 10^5^ cells per well). After reaching 80% confluence, cultures were transfected for 24 h with 50 nM of either the miR-199a-5p or negative control mimics. The endogenous levels of XIAP protein were measured using a standard immunoblot procedure. Briefly, total protein was extracted using mechanical detachment of the cells followed by lysis in RIPA lysis buffer supplemented with Complete EDTA-free protease inhibitor cocktail (Merck), incubated for 30 min at 4 °C and cleared by centrifugation (12.000× *g* for 10 min at 4 °C). Protein concentration of the lysates was quantified using the bicinchoninic acid method (ThermoFisher Scientific) following the manufacturer’s protocol. Cell lysates were mixed with Laemmli buffer (2mercaptoethanol, 0.1% (Sigma); bromophenol blue, 0.0005% (UBS Affimetrix; Santa Clara, CA, USA); Glycerol, 10%; Sodium dodecyl sulfate (SDS), 2% and Tris-HCl pH 6.8) and boiled for 5 min at 100 °C. After SDS-polyacrylamide gel electrophoresis (SDS-PAGE), proteins were transferred to polyvinylidene difluoride membranes (PVDF, Merk). Then, membranes were blocked with 5% non-fat milk diluted in TBS-T buffer (Tris buffer saline (Fischer Scientific) plus 0.05% (*v*/*v*) Tween20 (Merk) and incubated overnight at 4 °C with the appropriate specific antibodies diluted in blocking solution. Afterwards, blots were incubated at room temperature (RT) for 90 min with the correspondent horseradish peroxidase (HRP) conjugated secondary antibody (see Table 2) diluted in a blocking solution. Detection by enhanced chemiluminescence (ECL) was performed using SuperSignal West Pico chemiluminescent assay (Thermo Fisher Scientific) according to the manufacturer’s instructions. Blot images were acquired using ImageScanner III and LabScan v6.0 software (GE Healthcare Bio-Sciences AB; Chicago, IL, USA) and band intensities were measured using ImageJ software version 1.54f [77]. All employed antibodies recognized the specific band or bands of expected molecular weight for their target without detection of any non-specific bands.

### 4.8. Histology

We euthanized the animals at 0, 3, and 7 dpi by intraperitoneal injection of 500 mg/Kg sodium pentobarbital (Vetoquinol; Madrid, Spain) just before performing transcardial perfusion with saline, followed by 4% paraformaldehyde (Sigma) in 0.1 M phosphate buffer (PB), pH 7.4. We collected 1 cm long spinal cord samples centered around the injury site, which were immersed in 4% paraformaldehyde for 48 h at 4 °C and then cryoprotected in 30% sucrose in PB (*w*/*v*) until they sank. Subsequently, we embedded the samples in Tissue-Tek optimum cutting temperature compound (Sakura Finetek Europe B.V., Alphen aan den Rijn, The Netherlands) and froze them at −80 °C until further use. The tissue was sectioned into 20 μm thick transverse slices using an HM560 cryostat (Microm International GmbH, Walldorf, Germany) and mounted on superfrost slides (Thermo Fisher Scientific). Serial sections, separated by 300 μm, covered the injured segment along with the adjacent rostral and caudal segments.

### 4.9. Fluorescent In Situ Hybridization (FISH)

For FISH staining of miR-199a-5p in the spinal cord sections, we followed protocol by Søe and cols [78]. All solutions were prepared using autoclaved H_2_O-DEPC (diethylpyrocarbonate 1:1000 in distilled water; Sigma-Aldrich, Madrid, Spain). In brief, we thawed spinal cord sections and treated them with proteinase K for 15 min at 37 °C (40 µg/mL of proteinase K (Qiagen) diluted in EDTA (1 mM) and NaCl (1 mM) in Tris/HCl 40 mM, pH 7.4 buffer). To avoid non-specific ionic bindings, we incubated the sections in an acetylation buffer composed of triethanolamine (1.3% (*v*/*v*)), HCl (0.06% (*v*/*v*)), and acetic anhydride (0.25% (*v*/*v*)) for 10 min at RT. Then, we incubated the sections in hybridization buffer (1× miRCURY LNA miRNA ISH buffer; Qiagen) for 30 min at 65 °C before hybridizing them with miR-199a-5p or negative control (cel-miR-67) probes (Eurogentec; Seraing, Belgium). We designed both probes following Søe and cols. (see Table 3). We diluted probes to a final concentration of 200 nM in hybridization buffer 1×, denatured them for 5 min at 80 °C, and incubated them with the sections for 1 h at 65 °C. Then, we sequentially washed cells in 75 mM, 15 mM, and 1.5 mM saline–sodium citrate solutions (SSC; Fisher Scientific) for 3 min at 65 °C each and a final wash in 1.5 mM SSC for 3 min at RT. Then, we incubated the sections in blocking buffer (horse serum (5%) and BSA (1%) in PBS-T-DEPC (DEPC treated PBS with 0.1% Tween 20) for 15 min at 37 °C and then with an alkaline phosphatase-conjugated sheep-anti-digoxigenin antibody (see Table 2) for 20 min at 37 °C. Finally, to detect hybridization we incubated the slides with the alkaline phosphatase subtract Vector Blue (Vector Laboratories; Newark, CA, USA) following the manufacturer’s protocol.

### 4.10. Immunofluorescence

In vitro. C6 cells were cultured over 12 mm round glass coverslips inside a 24-well plate (10^4^ cells per well). After reaching 80% confluence, cultures were transfected for 24 h with 50 nM of either miR-199a-5p or negative control mimics. Then, cells were fixed with 4% paraformaldehyde for 20 min at RT and then permeabilized and blocked by incubation overnight at 4 °C with blocking buffer (5% goat serum (Merk) and 0.2% Triton X-100 (Merk) in PBS 1×). Samples were then incubated for 2 h at RT in a solution of anti-XIAP antibody (see Table 2) diluted in blocking buffer, followed by three washes in PBS and incubation in a solution of secondary antibody Alexa Fluor 488 nm-conjugated rabbit anti-goat (see Table 2), diluted in blocking buffer. Finally, coverslips were mounted on glass slides employing Fluorescence Mounting Medium (DAKO North America Inc. Agilent Technologies Inc.; Santa Clara, CA, USA) with the fluorescent probe of nucleic acids 4′,6-diamino-2-fenilindol (DAPI) for nuclei staining (Merck). Preparations were imaged in an epifluorescence microscope (DM5000B, Leica Microsystem GmbH; Wetzlar, Germany) with a 20× objective obtaining 5 images per sample. No XIAP staining was detected in controls without primary antibodies.

In vivo (the same procedure was performed after FISH assay). To carry out the immunofluorescence staining, sections were first heated at 37 °C for 45 min, rehydrated in phosphate-buffered saline, PBS (Thermo Fisher Scientific), and blocked and permeabilized by incubation for 2 h at RT in a solution composed by 5% (*v*/*v*) normal goat serum (Sigma Aldrich) and 0.2% (*v*/*v*) Triton X-100 (Sigma Aldrich) in PBS. Afterwards, sections were incubated overnight at 4 °C with specific primary antibodies diluted at appropriate concentrations (see Table 1) in blocking solution, rinsed in PBS, and incubated for 2 h at RT with the appropriate Alexa Fluor-conjugated secondary antibodies (see Table 1), also diluted in blocking solution. The stained sections were mounted with a Fluorescent Mounting Medium (Agilent Technologies Inc) containing the fluorescent marker of nucleic acids 4′,6-diamino-2-fenilindol, DAPI, 1.5 μg/mL (Sigma). Images of stained sections of the spinal cord were taken using a fully motorized Olympus IX83 microscope (Olympus, Tokio, Japan) equipped with Cell Sense Dimensions software V4.1.1 (Olympus).

### 4.11. Imagen Analysis

In vitro: Using the object classification tools included in the detection tools QuPath software vs. 0.4.3 [79], we detected all cells present in the image through DAPI nuclei staining, placing a 5 µm ellipsoidal diameter ROI on the cytoplasm of the chosen cells, and then measuring the mean intensity of Alexa 488 antibody in the corresponding image. We measured a total of 1500 cells per condition, with 5 images per experiment in three independent experiments.

In vivo: Following measurement of miR-199a-5p and XIAP protein in neurons and oligodendrocytes, we acquired images of the whole rat spinal cord sections through the macro to micro function of an IX83 scanR microscope using a 10× objective at high magnification and the deep learning-based image analysis approach TruAI integrated CellSens Dimensions software V4.1.1 that uses deep convolutional neural network architecture for object segmentation. During the training phase, we fed the network with nearly 1000 manually segmented neurons or oligodendrocytes across the gray/white matter of control and injured spinal cords. The background data surrounding each neural cell, as well as artifacts, were also identified. This training phase was carried out using the Deep Learning module operating under the Standard Network configuration and the Olympus protocols on 300,000 iterations with 5 checkpoints every 60,000 iterations. Although predictions by TruAI can be very precise and robust, the generated neural network was validated using the Olympus CellSens imaging software V4.1.1 to ensure that no artifacts or other errors were produced. A minimum of 85% congruence between manual and neural network identifications was set.

Once the neural network had been trained and validated, it was applied to all images to assign the probability of being part of a neuronal or oligodendrocytes nucleus to every pixel in the image. To identify neuronal nuclei, we considered only those particles composed of pixels above 50% probability and an area above 25 µm^2^.

### 4.12. Data Analysis

All data are expressed as means ± SD, as indicated in figure legends. Statistical significance of the treatment effects was tested using the paired or non-paired Student’s *t*-test or the analysis of variance test (ANOVA) followed by Tukey Multiple Comparison post hoc test, depending on the characteristics of the data. Normality and homoscedasticity of the data were assessed using Shapiro–Wilk and Bartlett tests, respectively, using the shaphiro.test and bartlett.test functions of R software V4.2.2 [80]. Statistical analyses and graphics were carried out and made using Prism Software 5.0 (GraphPad Software Inc., Insight Partners, New York, NY, USA) and R statistical language. Differences were considered statistically significant when the *p*-value was <0.05.

## 5. Conclusions

In this study, we first validated that miR-199a-5p regulates the anti-apoptotic protein XIAP. We also demonstrated that miR-199a-5p expression is upregulated following SCI and is heterogeneously expressed in both gray and white matter. These findings offer new insights into the roles of miR-199a-5p and XIAP in apoptotic cell death after SCI, which may be valuable for developing therapeutic strategies for SCI treatment.

## Figures and Tables

**Table 1:** Primers used for subcloning of XIAP 3′UTR-wt and XIAP 3′UTR-mut, and DNA sequencing.

Primers	Sequences (5′-3′)
**XIAP 3′UTR-wt**	*Forward:* ATCGAGCTCCACAGTAGGCATGTTATG *Reverse*: ATAGTCGACCTGTGATGCTTTTCTATGTCAG
**XIAP 3′UTR-mut**	*Forward*: GTTCCAAGATCTTTGGAGG *Reverse*: CCTCCAAAGATCTTGGAACAGTTC
**pmiRGLO sequencing**	CAAGAAGGGCGGCAAGATCG

**Table 2:** Detailed data for each antibody can be accessed at antibodyregistry.org using the provided RRID codes.

Antibodies		Reference
**Primary antibodies for Immunoblot**		
anti-XIAP		R&D Systems (Minneapolis, MN, USA) Cat# AF8221, RRID:AB_2215008
Anti-ß-Actin		BD Bioscience (Franklin Lakes, NJ, USA) Cat# 612656, RRID:AB_2289199
**Primary antibodies for Immunofluorescence**		
anti-XIAP		In vitro: R&D Systems Cat# AF8221, RRID:AB_2215008
		In vivo: Abcam (Cambridge, UK) Cat# ab21278, RRID:AB_446157
anti-Neuronal Nuclei protein (NeuN)		Millipore (Burlington, MA, USA Cat# ABN78, RRID:AB_10807945
anti-Neuronal Nuclei protein clon 60 (NeuN)		Merck Cat# MAB377, RRID:AB/11210778
anti-Adenomatous Polyposis Protein (APC)		Millipore Cat# OP80, RRID:AB_2057371
anti-Placental gluthatione S-transferase (GST-pi)		MBL International (Woburn, MA, USA) Cat# 311-H, RRID:AB_591790
anti-Glial Fibrillary Acidic Protein (GFAP)		Abcam Cat# ab4674, RRID:AB_304558
**Primary antibodies for FISH**		
Alkaline phosphatase-conjugated sheep-anti-digoxigenin	Roche Cat# 11093274910, RRID:AB_2734716	
**Secondary antibodies for Immunoblot**		
HRP-conjugated goat anti-rabbit	Cell Signaling Technology (Danvers, MA, USA)Cat#7074, RRID:AB_2099233	
HRP-conjugated goat anti-mouse	Cell Signaling Technology Cat#7076, RRID:AB_330924	
**Secondary antibodies for Immunofluorescence**		
Alexa Fluor 488 goat anti-rabbit	Life Technologies Cat# A11034, RRID:AB_10562715	
Alexa Fluor 488 goat anti-mouse highly cross adsorbed	Molecular Probes (Eugene, OR, USA) Cat# A11029, RRID:AB_138404	

**Table 3:** Sequences of the probes used for miR-199-5p detection by FISH. Oligonucleotides probe sequences complementary to miR-199a-5p and negative control mimics with no complementary targets. Probes are composed of the combination of 2′-O-methyl ([]) and LNA ({ }) nucleotides designed following the method from Søe et al. [78].

Probe	Sequence
**Negative Control**	5′-DIG-{G}[T]{GU}[A]{AC}[A]{CG}[T]{CU}[A]{UA}[C]{GC}[C]{CA}-3′
**miR-199a-5p**	5′-DIG-{G}[AA]{C}[AG]{G}[UA]{G}[UC]{T}[GA]{A}[CA]{C}[UG]{GG}-3′

## Data Availability

Data is contained within the article and supplementary materials.

## Supplementary Materials

The following supporting information can be downloaded at: https://www.mdpi.com/article/10.3390/ijms252212374/s1.
