# Supplementary material for: Identification of a New Role of miR-199a-5p as Factor Implied in Neuronal Damage: Decreasing the Expression of Its Target X-Linked Anti-Apoptotic Protein (XIAP) After SCI
Source: Int J Mol Sci. 2024 Nov 18;25(22):12374. doi: 10.3390/ijms252212374 (PMC11594351; doi:10.3390/ijms252212374)
Supplement: Supplementary file 1 [file ijms-25-12374-s001.zip › ijms-3268829-supplementary.pdf]

# Supplementary Figure S1

Data from previous RT-qPCRs studies of miR-199a-5p levels after SCI

| | | Ctrl | 3 dpi | 7 dpi |
|---|---|---|---|---|
| **Yunta et al., 2012** | **raw data±SD** | 7.83±0.13 | 8.62±0.49 | 8.89±0.52 |
| | statistics | | | 1.5E-02 |
| **Chen et al., 2022** | **raw data±SD** | 1867.89±262.17 | 1881.33±552.14 | |
| | statistics | | 3.329 | |
| **Liu et al., 2009** (Fold change = log2(SCI/ SHAM)) | **raw data±SD** | - | - | 1.02±0.97 |
| | statistics | | | 2.2E-01 |


Yunta, Mónica, Manuel Nieto-Díaz, Francisco J. Esteban, Marcos Caballero-López, Rosa Navarro-Ruíz, David Reigada, D. Wolfgang Pita-Thomas, Ángela del Águila, Teresa Muñoz-Galdeano, and Rodrigo M. Maza. 2012. MicroRNA Dysregulation in the Spinal Cord following Traumatic Injury. *PLOS ONE* 7. Public Library of Science: e34534. https://doi.org/10.1371/journal.pone.0034534.

Chen, Jia-Nan, Yi-Ning Zhang, Li-Ge Tian, Ying Zhang, Xin-Yu Li, and Bin Ning. 2022. Down-regulating Circular RNA Prkcsh suppresses the inflammatory response after spinal cord injury. *Neural Regeneration Research* 17: 144–151. https://doi.org/10.4103/1673-5374.314114.

Liu, Nai-Kui, Xiao-Fei Wang, Qing-Bo Lu, and Xiao-Ming Xu. 2009. Altered microRNA expression following traumatic spinal cord injury. *Experimental Neurology* 219: 424–429. https://doi.org/10.1016/j.expneurol.2009.06.015.

## Supplementary Figure S2

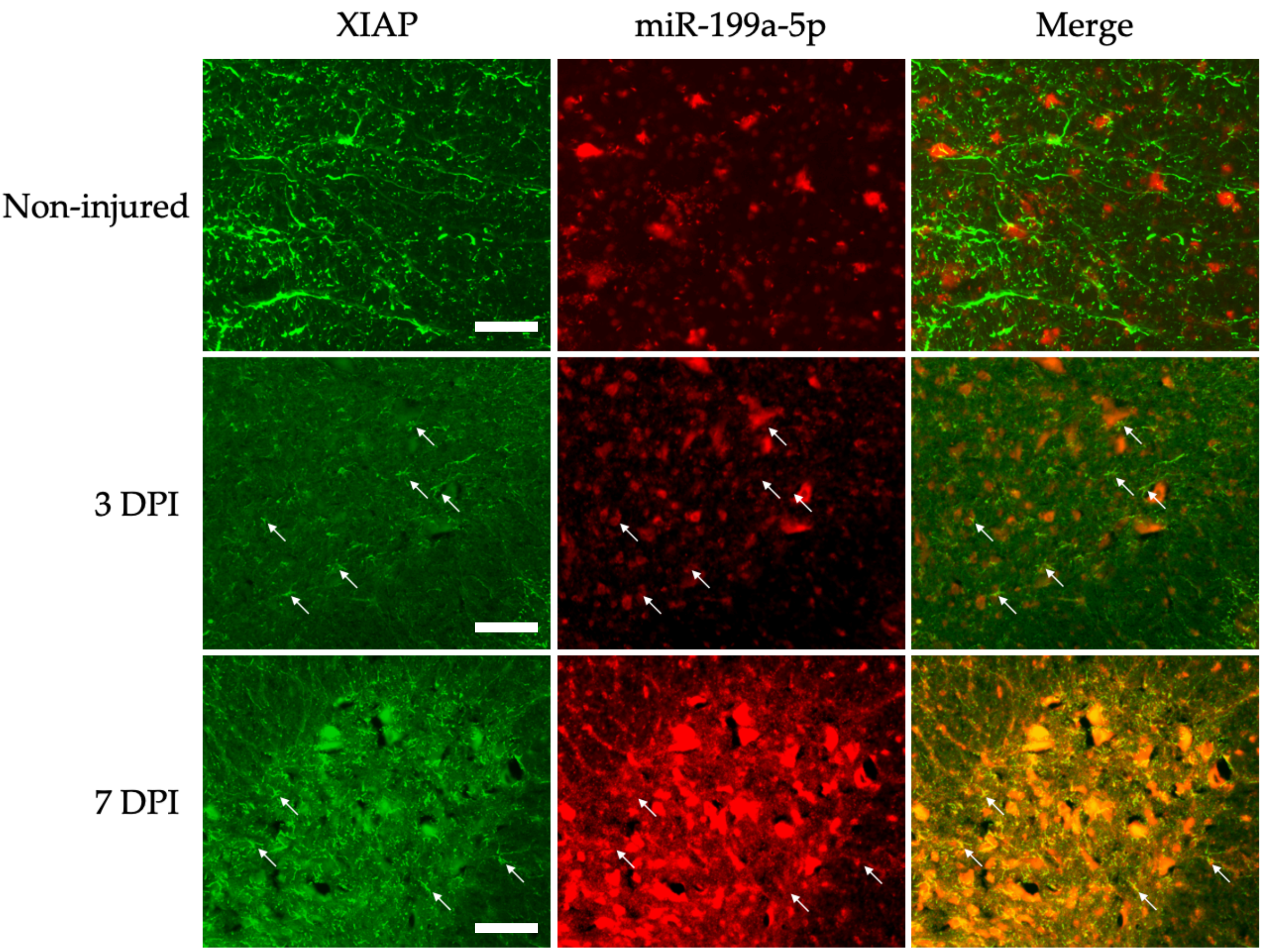


**miR-199a-5p is Not Expressed is Astrocytes after SCI:** High-magnification representative images from FISH/IF assays performed on coronal sections of control (top row) and post-injury (3 days, middle row; 7 days, bottom row) rat spinal cords, co-labeled with astrocytes.specific GFAP antibody (green, left) and the miR-199a-5p probe (red, middle), with merge images shown on the right. Scale bar: 50 µm. White arrow indicate GFAP-positive astrocytes lacking miR-199a-5p co-labeling, suggesting astrocytes are not positive for miR-199a-5p expression. Due to the absence of co-labeling between astrocytes and miR-199a-5p, this cell type was excluded from further analysis in this study
